# Early Exposure of Infants to GI Nematodes Induces Th2 Dominant Immune Responses Which Are Unaffected by Periodic Anthelminthic Treatment

**DOI:** 10.1371/journal.pntd.0000433

**Published:** 2009-05-19

**Authors:** Victoria J. Wright, Shaali Makame Ame, Haji Said Haji, Rosemary E. Weir, David Goodman, David I. Pritchard, Mahdi Ramsan Mohamed, Hamad Juma Haji, James M. Tielsch, Rebecca J. Stoltzfus, Quentin D. Bickle

**Affiliations:** 1 Department of Infectious and Tropical Diseases, London School of Hygiene and Tropical Medicine, London, United Kingdom; 2 Public Health Laboratory Ivo de Carneri, Wawi, Chake Chake, Pemba Island, Zanzibar, United Republic of Tanzania; 3 Centre for Human Nutrition, Department of International Health, The Johns Hopkins Bloomberg School of Public Health, Baltimore, Maryland, United States of America; 4 School of Pharmacy, University of Nottingham, Nottingham, United Kingdom; 5 RTI International, Dar es Salam, United Republic of Tanzania; 6 Division of Nutritional Sciences, Cornell University, Ithaca, New York, United States of America; Sabin Vaccine Institute, United States of America

## Abstract

We have previously shown a reduction in anaemia and wasting malnutrition in infants <3 years old in Pemba Island, Zanzibar, following repeated anthelminthic treatment for the endemic gastrointestinal (GI) nematodes *Ascaris lumbricoides*, hookworm and *Trichuris trichiura*. In view of the low intensity of worm infections in this age group, this was unexpected, and it was proposed that immune responses to the worms rather than their direct effects may play a significant role in morbidity in infants and that anthelminthic treatment may alleviate such effects. Therefore, the primary aims of this study were to characterise the immune response to initial/early GI nematode infections in infants and the effects of anthelminthic treatment on such immune responses. The frequency and levels of Th1/Th2 cytokines (IL-5, IL-13, IFN-γ and IL-10) induced by the worms were evaluated in 666 infants aged 6–24 months using the Whole Blood Assay. *Ascaris* and hookworm antigens induced predominantly Th2 cytokine responses, and levels of IL-5 and IL-13 were significantly correlated. The frequencies and levels of responses were higher for both *Ascaris* positive and hookworm positive infants compared with worm negative individuals, but very few infants made *Trichuris*-specific cytokine responses. Infants treated every 3 months with mebendazole showed a significantly lower prevalence of infection compared with placebo-treated controls at one year following baseline. At follow-up, cytokine responses to *Ascaris* and hookworm antigens, which remained Th2 biased, were increased compared with baseline but were not significantly affected by treatment. However, blood eosinophil levels, which were elevated in worm-infected children, were significantly lower in treated children. Thus the effect of deworming in this age group on anaemia and wasting malnutrition, which were replicated in this study, could not be explained by modification of cytokine responses but may be related to eosinophil function.

## Author Summary

Infants and very young children commonly become infected with intestinal nematode infections. However, the worm burdens are generally very light, so a beneficial effect of deworming on wasting malnutrition and anaemia in this age group which we have demonstrated was unexpected and the mechanism unclear. To investigate this, we have, for the first time, determined whether such worm infections in infants induce significant immune reactions which might be detrimental to nutrition and growth e.g. by inducing inflammation in the gut or by cytokine effects on erythropoiesis. We also determined if such responses are modulated by regular deworming over a 9 month period. Peripheral blood cells from infants infected with *Ascaris* and hookworms in particular responded to stimulation with worm antigens, producing predominantly Th2 cytokines. Although the Th2 cytokine responses in the periphery were not significantly altered by deworming, the levels of eosinophils, which are regulated by the Th2 cytokine, IL-5, were lower after treatment. It is possible that eosinophils play a role in gut pathology leading to wasting malnutrition and anaemia in the very young and that this effect is reduced by deworming.

## Introduction

In endemic countries infants are exposed to gastrointestinal (GI) nematode infections soon after birth and infection intensity increases during childhood. Helminth infections in children are associated with malnutrition [1],[2], linear growth stunting [3] as well as iron deficiency anaemia [4],[5], effects related to the intensity of infection [6] and generally attributed to the direct and indirect effects of the worms on the gut i.e. blood loss, mucosal damage, secondary infection, malabsorption [7]–[10]. Several studies have now documented that deworming leads to improvements in nutritional outcomes e.g. anaemia and wasting malnutrition and in development of school-aged children who often harbour the highest intensities of these worms [11]–[13]. However, a recent study in Pemba found significant benefits of anthelminthic treatment on growth, anaemia and appetite in children <30 months of age who harboured very light GI nematode infections [14]. Indeed the benefits of mebendazole were significant only in this younger age group and not in children 30–71 months old. In view of this unexpected effect of deworming very lightly infected children, it was suggested that the nutritional benefit may be related to prevention of the indirect effects of the worms such as on the immune responses they induce rather than to their direct effects.

Various aspects of the immune response to the initial/early exposure to GI nematodes might contribute to anaemia and malnutrition, and this may be alleviated by worm treatment. Pro-inflammatory cytokines and acute phase proteins can suppress appetite [15],[16], induce protein loss [17] and raise the levels of resting energy expenditure [18],[19], as well as affect anaemia (the anaemia of chronic disease) [15]–[17],[19],[20]. One possibility therefore is that primary exposures to GI nematode infections induce inflammatory (Th1-mediated) responses in a significant proportion of the infants resulting in anaemia and malnutrition. Although older humans in endemic areas generally develop Th2 dominated cytokine responses to GI nematode infections, characterised functionally by IgE and eosinophilia [21]–[23], some studies have shown that Th1 cytokines are induced [24]. The responses to initial exposures in infants has not been studied previously but studies of gut nematode infections in mice have shown that, depending on the worm species and host genotype, the response to primary infection can be polarized to either Th1 or Th2 [25] and influenced by infection intensity [26]. By analogy, it may be hypothesised that light primary exposures in the human population may result in Th1 responses, in at least a proportion of individuals.

Th2-mediated responses may also affect gut function leading to impaired nutrition. Studies of GI nematode infections in mice have shown that the barrier function of the mucosa can be profoundly altered by the action of Th2 cytokines on epithelial cells and/or mast cells resulting in increased mucosal permeability, reduced glucose absorption, increased ion secretion and intra-luminal fluid accumulation [27]–[30]. Infections of *Ascaris suum* in pigs, which are considered a relevant model for human ascariasis, cause similar effects coincident with upregulated expression of Th2 cytokines [31]. In humans, *T. trichiura* can induce mast cell infiltration and an immediate hypersensitivity response in the colon of infected children leading to release of histamine [32]. During *T. suis* infection in pigs, a good model for human trichuriasis, upregulation of expression of Th2 cytokines in the mucosa parallels mucosal hypertrophy characterised by infiltration of mast cells and eosinophils which may play an immunopathological role [33]. Similarly in humans, hookworm infections result in eosinophil infiltration [34], Charcot-Leyden crystal production [35] and, following infection with *Ancylostoma caninum*, eosinophilic enteritis [36]. Worm-induced Th2 cytokines can also induce increased smooth muscle contractility in mice [28],[37],[38] and pigs [31] and, in mice, can induce epithelial cell turnover [39], goblet cell hyperplasia and mucus secretion [40],[41].

The current immunological study was a trial within a larger field based randomised treatment trial (manuscript in preparation) to confirm the beneficial effects of treating intestinal helminth infections in early childhood on anaemia and malnutrition [14]. The immunological study was a primary aim of the project designed to investigate possible immunological mechanisms involved in the pathogenesis of these early infections and the amelioration of this by deworming. The specific aims were to establish (i) if measurable immune responses to worm infections (cytokines, acute phase proteins) could be demonstrated in very young (6–24 months) children harbouring light infections (ii) if so, whether such infants made predominantly Th1 or Th2 cytokine responses or some one and some the other; (iii) whether such responses were altered by periodic (3-monthly) anthelminthic treatment which might explain the benefits afforded by such treatment in this age group.

## Materials and Methods

This study was nested within a community-based treatment trial designed to test whether periodic mebendazole treatment in 6–24 month old infants would decrease rates of severe anaemia and protein-energy malnutrition (International Standard Randomised Controlled Trial Number 83988447). The study was performed between September 2003 and October 2004 at the Public Health Laboratory-Ivo de Carneri, Pemba Island, Zanzibar, United Republic of Tanzania. Pemba Island is densely populated and mostly rural, with subsistence farming as the main economic activity. *Plasmodium falciparum* malaria is holoendemic, as are the geohelminths, *Ascaris lumbricoides*, *Trichuris trichiura*, *Ancylostoma duodenale*, and *Necator americanus*.

### Study participants

Initially 2664 children aged 6–23 months were screened for helminth infection. During the screening process, age-matched triplets of infants (comprising 2 infected (matched for infection species) and 1 uninfected infant) were formed and randomised for treatment stratified by age (3 groups 6–11, 12–17 and 18–23 months) and by infection status (*Ascaris*, *Trichuris*, *Ascaris* and *Trichuris*, hookworm with or without any other infection). These children formed the immunology study cohort; 335 infants were randomised to placebo and 318 to mebendazole treatment. The CONSORT protocol is in Figure S1 and Protocol S1. All children screened but not selected for the immunology study were subsequently randomised in the main community-based treatment trial with random allocation to treatment or placebo groups. The immunology study children were still involved in the randomised treatment trial (manuscript in preparation). Age-matched selection into the immunology study was essential to the design, because the probability of infection was very strongly related to infant age. Without age-matched selection, the infected children would naturally have been older than the uninfected children, creating a biased comparison with regard to infection status. Having created the age-matched samples of infected and non-infected children, we then analysed the data without regard to the original matching. This is a valid approach for matched follow-up (cohort) studies [42]. At baseline blood was taken for the immunological investigations after which the infants were treated with a 3 day course of mebendazole, 100 mg twice daily, or identical placebo that was repeated every 3 months over a study period of 12 months. A blood sample was again taken for immunological studies 1 month after the 3^rd^ treatment round to allow time for any possible effects of worm reductions on cytokine responses to develop. The study was approved by the ethical review committees of the London School of Hygiene and Tropical Medicine, Johns Hopkins Bloomberg School of Public Health, Cornell University, and the Ministry of Health of Zanzibar. Because of the high rate of illiteracy amongst parents, verbal informed consent was obtained from the mothers or from the guardians of all enrolled infants, documented by signature of a literate witness, following the ethical review committees' approval.

### Parasitology

Stool samples were collected on 2 consecutive days and stored at 4°C. Individual Kato-Katz slides were prepared from both samples and the means taken [43]. The two samples were then combined and 2 g were used for assessment by a sedimentation technique [44]. In a small proportion of the *Ascaris* positive stool samples (6.6%), the *Ascaris* egg counts were very high and egg counts in individual Kato-Katz slides were stopped at 999 (i.e. 23, 976 epg). The percentage of egg reduction induced by treatment (ERR) was estimated as 100[1−exp(−D)]%], where D was the mean difference for a particular treatment.

### Antigen preparation

The somatic hookworm antigen from adult *Necator americanus*, maintained in a hamster life cycle was prepared as described elsewhere [35]. *Necator americanus* worms were kindly provided by Prof. J Behnke, Prof. D Pritchard and Dr A Brown of Nottingham University. The *Trichuris suis* and *Ascaris suum* were kindly provided by Dr Dolores Hill and Dr Joseph Urban Jnr of the United States Department of Agriculture. The *Ascaris* and *Trichuris* antigens were prepared as described elsewhere [45],[46]. In brief, the *Ascaris* antigen was derived from adult *A. suum* that were homogenized, extracted in 1× Dulbecco's PBS overnight at 4°C, spun at 20,000 g, concentrated and dialysed against 10 mM TBS. The supernatant was filter-sterilised, aliquoted and stored at −80°C. Adult *T. suis* worms were cultured for 36 hours and culture fluid used for the ES antigen. Somatic antigen from *T. suis* was prepared as for *A. suum* above. Protein concentrations were determined using the Bio-Rad protein assay.

### Whole blood culture

The Whole Blood Assay (WBA) was carried out as described elsewhere [35]. Heparinised venous blood was used no later than 4 hours after venepuncture. Preliminary validation of the helminth antigens to induce recall cytokine responses in the WBA was carried out in endemic helminth infected teenagers before use in the infant studies. *Ascaris* and *Trichuris* antigens were used at a final concentration of 30 µg/ml, whilst a pool of somatic hookworm antigens was used at 20 µg/ml. Phytohemagglutinin and purified protein derivative concentrations as well as haematology procedures for differential cell counts were carried out as described elsewhere [35].

### Cytokine ELISA

Culture supernatants were stored at −80°C. Matched monoclonal antibody pairs from Pharmingen (Oxford, UK) or R & D Systems (Abingdon, UK) were used according to the manufacturer's instructions (IL-5: TRFK5 and JES1-5A10, IL-10: JES3-9D7 and JES3-12G8, IL-13: JES10-5A2 and B69-2 from Pharmingen; IFN-γ from R & D Systems). When a new kit was introduced it was validated by testing in parallel with the previous kit using a large batch of positive culture supernatant which was used throughout the study.

### Statistical analysis

All analyses were performed using the STATA statistical analysis software package (version 9; Stata Corp). Medium alone negative control values were subtracted from all results that were above the lower limit of assay detection (i.e. 15 pg/ml). All ELISA plate readings were standardised for each cytokine by use of the positive control supernatant run on each plate in duplicate. Where data are presented as percentage responders, a response was defined as a cytokine concentration of >31.24 pg/ml, derived from a comparison of the frequency of responses in worm negative infants with worm positive infants, where a bimodal distribution was observed. Contingency tables and Pearson's Chi-squared tests were used to compare proportions of responders. Fisher's exact test was used for small sample sizes. T test or ANOVA were used with adjustment for multiple comparisons by Bonferroni procedure. Effects of age and sex were investigated using ANOVA. Also in view of the report of effects of malaria infection on helminth cytokine responses [24], malaria infection status was included in the analysis. Non-normally distributed variables were transformed, or non-parametric tests (Mann Whitney test, Wilcoxon signed rank test or Kruskal-Wallis test) were used. To examine the relationships between two variables non-parametric regression (Lowess) was used. If the relationship was approximately linear, Pearson correlation coefficients were calculated and a linear regression model was fitted. If the relationship was non-linear, the data were transformed. If this failed to produce a linear relationship, then the non-parametric correlation Spearman test was used. Bootstrap was used to infer variances of regression coefficients, P-values and 95% confidence intervals when the data were not normal. Regression models were used to identify predictors for cytokine response adjusting for age, sex and malaria. Cure rates, percentage reduction in prevalence and egg reduction rates were calculated as described [47]. Cytokine responses measured from stimulation of whole blood with *Trichuris* ES and somatic antigen were minimal in infants, and mean response to the two antigens were used in analysis.

## Results

### Parasitology at baseline

The 666 subjects were selected on the basis of being (any) worm positive or negative at a ratio of approximately 2∶1 (70.7% were worm positive) with age-matching of infected and uninfected infants. Amongst the worm positive infants, 42.0% had *Ascaris* infection (±other worms), 16.8% were positive for hookworm (±other worms) and 71.5% were positive for *Trichuris* (±other worms) (Table 1 and Figures S2, S3, and S4). Mean intensities (eggs per gram [epg]) were 1061 for *Ascaris*, 213 for hookworm and 213 for *Trichuris* (see also Figure S5) and there was no significant effect of co-infection with one of the other worms on intensities of infection. According to WHO categorisation [48] the majority of the infants harboured “light” infections (80.4% *Ascaris*, 1–4999 epg, 96.8% hookworm, 1–1999 epg, 89.9% *Trichuris*, 1–999 epg). Egg counts were significantly positively associated with age for *Ascaris* egg positive infants (n = 184, β = 1.06, P = 0.047, 95% CI 1.00–1.12) but this was not significant for *Trichuris* or hookworm egg positive infants. There was no significant association between intensity and sex. The prevalence of *Plasmodium* species infection in this age group was 24.9%.

**Table 1:** Demographic and parasitological characteristics of subjects at baseline.

Infection group		N (%)	Sex (M∶F)	Age mean, months (95% CI)	Malaria infection (%)	N	Infection intensity (epg, mean (95%CI)
**Worm negative**		195 (29.3)	107∶88	15.6 (15.0–16.1)	24.6	0	
	Pl	101	50∶51	15.7 (14.9–16.5)	30.7	0	
	Mbz	94	57∶37	15.4 (14.5–16.2)	18.1	0	
***Ascaris*** ** positive**		198 (42.0)	96∶102	15.9 (15.3–16.5)	22.3	184*	1060.8 (840.1–1339.5)
	Pl	97	50∶47	16.07 (15.2–16.9)	18.6	94	1224.9 (891.6–1682.6)
	Mbz	96	42∶54	15.5 (14.7–16.3)	26.0	90	912.8 (645.7–1290.43)
**Hookworm positive**		79 (16.8)	41∶38	17.0 (16.1–18.0)	26.7	63*	213.2 (161.4–281.5)
	Pl	37	17∶20	17.1 (15.7–18.5)	21.6	32	187.9 (126.7–278.6)
	Mbz	38	22∶16	16.9 (15.5–18.4)	31.6	31	242.9 (161.1–366.2)
***Trichuris*** ** positive**		337 (71.5)	170∶167	17.5 (17.1–18.0)	27.3	308*	212.8 (186.8–242.5)
	Pl	169	86∶83	17.7 (17.1–18.2)	24.3	162	212.4 (177.4–254.2)
	Mbz	157	77∶80	17.3 (16.7–17.9)	30.6	146	213.3 (176.1–258.4)

NOTE: CI, confidence interval. epg – eggs per gram of faeces (from duplicate Kato-Katz); values were log transformed and the geometric mean is presented. M, male F, female.

*: The numbers of infants contributing to intensity determination was lower than for prevalence since the latter was based on Kato Katz plus stool sedimentation whereas intensity was based on Kato Katz alone.

### Exposure of infants to GI nematode infections induces predominantly Th2 cytokine responses

**Figure 1:**
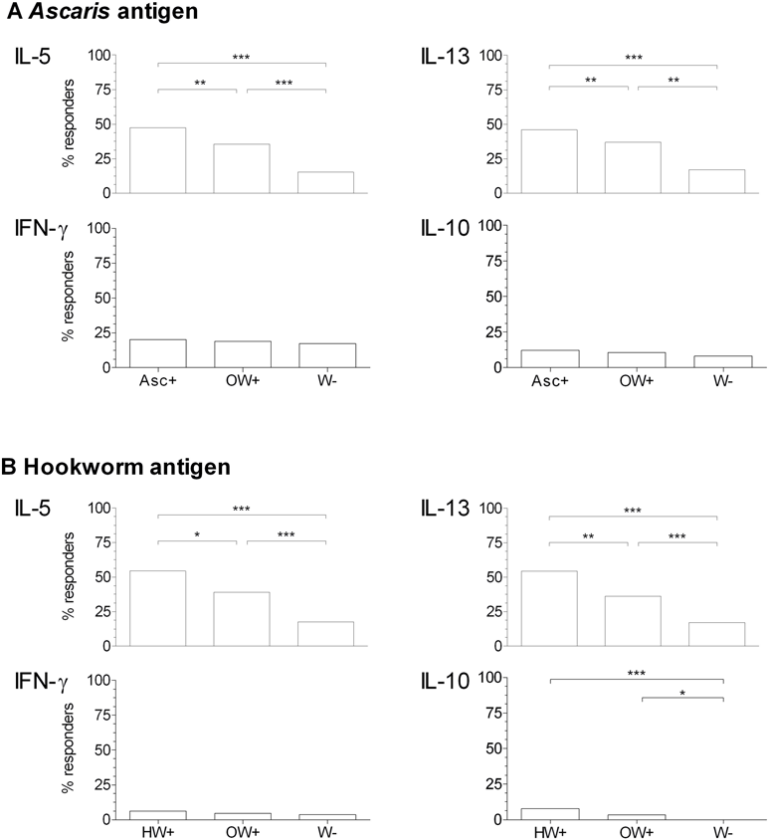
Frequency of IL-5, IL-13, IFN-γ & IL-10 responses in infants by infection status. Percentage of IL-5, IL-13, IFN-γ and IL-10 responders (cytokine responses >31.24 pg/ml) to:- A) *Ascaris* antigen in *Ascaris* positive infants (Asc+, n = 198); *Ascaris* negative/other worm positive infants (OW+, n = 273); and worm negative infants (W−, n = 195) or B) hookworm antigen in hookworm positive infants (HW+, n = 79); hookworm negative/other worm positive infants (OW+, n = 312); and worm negative infants (W−, n = 164). * P = 0.01–0.05, ** P = 0.001–0.01, *** P<0.001.

The frequency of subjects making cytokine responses above the cut off (i.e. >31.24 pg/ml, see Materials and Methods) at baseline for the *Ascaris*, and *Necator* antigens is shown in Figure 1.

Amongst *Ascaris* egg positive infants, 47% and 46% respectively made IL-5 and IL-13 responses whilst only 20% and 12% respectively made IFN-γ and IL-10 (Figure 1A). A significantly higher proportion of infants in the *Ascaris* positive group made IL-5 and IL-13 responses compared to both worm negative infants and *Ascaris* negative/other worm positive infants. The percentage of IL-5 and IL-13 responders was also significantly higher in *Ascaris* negative/other worm positive infants compared to worm negative infants. The percentage of IFN-γ or IL-10 responders was similar between the infection groups.

Similarly, 54% of hookworm egg positive infants made IL-5 and IL-13 responses to the homologous *Necator* antigen but only 6% and 7% made IFN-γ and IL-10 responses respectively (Figure 1B). A significantly higher percentage of hookworm positive infants made antigen-specific IL-5 and IL-13 positive responses compared to both worm negative infants and to hookworm negative/other worm positive infants. Hookworm negative/other worm positive infants also had a significantly higher percentage of responders compared to worm negative infants. The percentage of IFN-γ and IL-10 responders was very low in all groups.

There were minimal cytokine responses to the *Trichuris* antigen amongst the infants, with a very low percentage of responders (<5%), similar between the infection groups (data not shown).

The frequency of responses to phytohemagglutinin (PHA) and purified protein derivative (PPD) which were included in all assays were: IL-5, 85%; IL-13, 91%; IFN-γ, 52% and IL-10, 56% for PHA and the responses amongst BCG-vaccinated infants (BCG scar-positive) to PPD were:- IL-5, 48%, IL-13, 50%; IFN-γ, 78% and IL-10, 24%.

**Figure 2:**
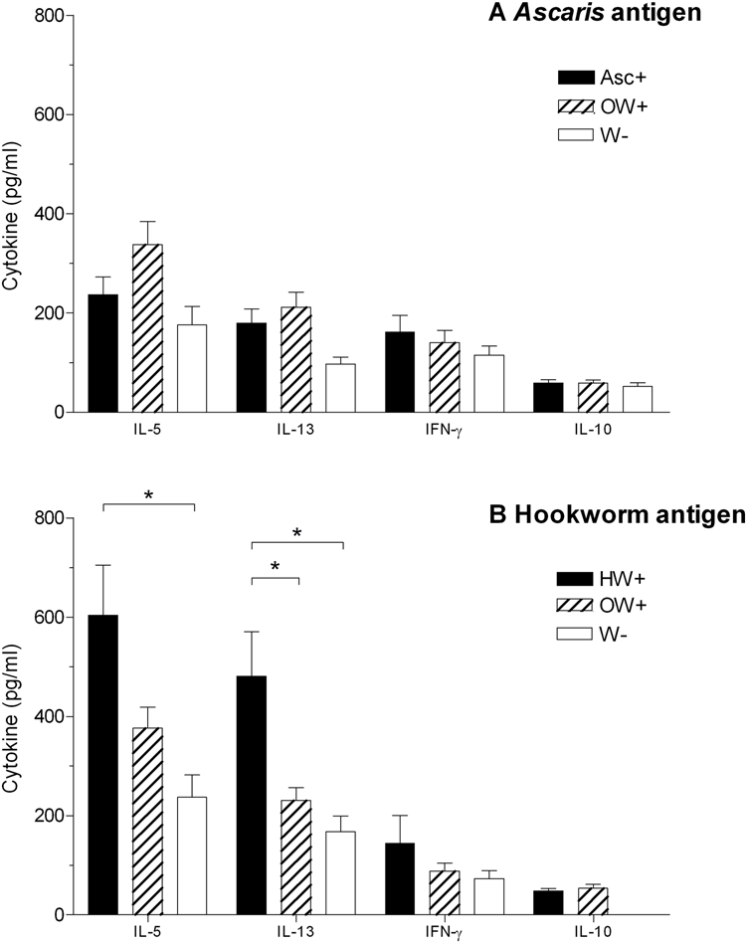
Mean level of IL-5, IL-13, IFN-γ and IL-10 responses in infants by infection status. Mean levels of cytokine responses (+SEM) amongst responders (responses >31.24 pg/ml) to:- A) *Ascaris* antigen in *Ascaris* positive (Asc+) infants ( n = 94, 91, 40 and 24, respectively, for IL-5, IL-13, IFN-γ, IL-10); *Ascaris* negative/other worm positive (OW+) infants (n = 97, 101, 52 and 29, respectively, for IL-5, IL-13, IFN-γ, IL-10); and worm negative (W−) infants (n = 30, 33, 34 and 16, respectively, for IL-5, IL-13, IFN-γ, IL-10) or B) hookworm antigen in hookworm positive (HW+) infants (n = 43, 43, 5 and 6, respectively, for IL-5, IL-13, IFN-γ, IL-10); hookworm negative/other worm positive (OW+) infants (n = 122, 113, 15 and 10, respectively, forIL-5, IL-13, IFN-γ, IL-10) and worm negative (W−) infants n = 29, 28, 6 and 0 respectively for IL-5, IL-13, IFN-γ, IL-10) * P = 0.01–0.05.

The mean cytokine concentrations produced in cultures to the helminth antigens for responders (>31.24 pg/ml) are shown in Figure 2 and reflect the data on frequency of responses.

The mean levels of IL-5 and IL-13 to *Ascaris* antigen were higher albeit not significantly in both *Ascaris* positive and *Ascaris* negative/other worm positive infants compared to worm negative infants (Figure 2A). The mean levels of IL-5 and IL-13 to hookworm antigen were significantly different between the infection groups (IL-5: ANOVA F(4, 189) = 4.58 P = 0.011, IL-13: ANOVA F(4, 179) = 5.98 P = 0.003). Mean IL-5 and IL-13 levels in the hookworm positive responders were significantly higher compared to worm negative infants (IL-5: P = 0.023, IL-13: P = 0.027), and IL-13 levels for the hookworm positive responders were also significantly higher compared to hookworm negative/other worm positive infants (P = 0.011) (Figure 2B). The mean responses to *Trichuris* antigen (<200 pg/ml) were not significantly different between the different infection groups (data not shown).

### Different Th2 cytokine responses were positively correlated and there was not a subset of infants making a predominantly Th1 response

The above data showed that Th2 cytokine responses to *Ascaris* and hookworm antigens predominate amongst infected infants. To demonstrate the reliability of the IL-5 and IL-13 results as an indicator of overall Th2 responsiveness we plotted the correlation between the levels of these two cytokines for the *Ascaris* antigen stimulations. IL-5 and IL-13 responses to *Ascaris* antigen in worm positive infants have a significant positive association (n = 471, repetitions = 1000, Bootstrap coefficient = 1.31, SE = 0.11, P<0.001, 95% CI 1.09–1.53, r^2^ = 0.8438). A similar correlation holds for hookworm antigen responses (data not shown).

Although there was no significant association between Th2 (IL-5 and IL-13) responses and IFN-γ responses in worm positive infants, the relatively few infants who made elevated IFN-γ responses to *Ascaris* and hookworm antigens also made elevated Th2 responses, whilst many infants who did not make IFN-γ responses made high IL-5 responses. Thus there was not a subset of infants who made a Th1 biased response.

### Level of cytokine response in relation to age and infection status

**Figure 3:**
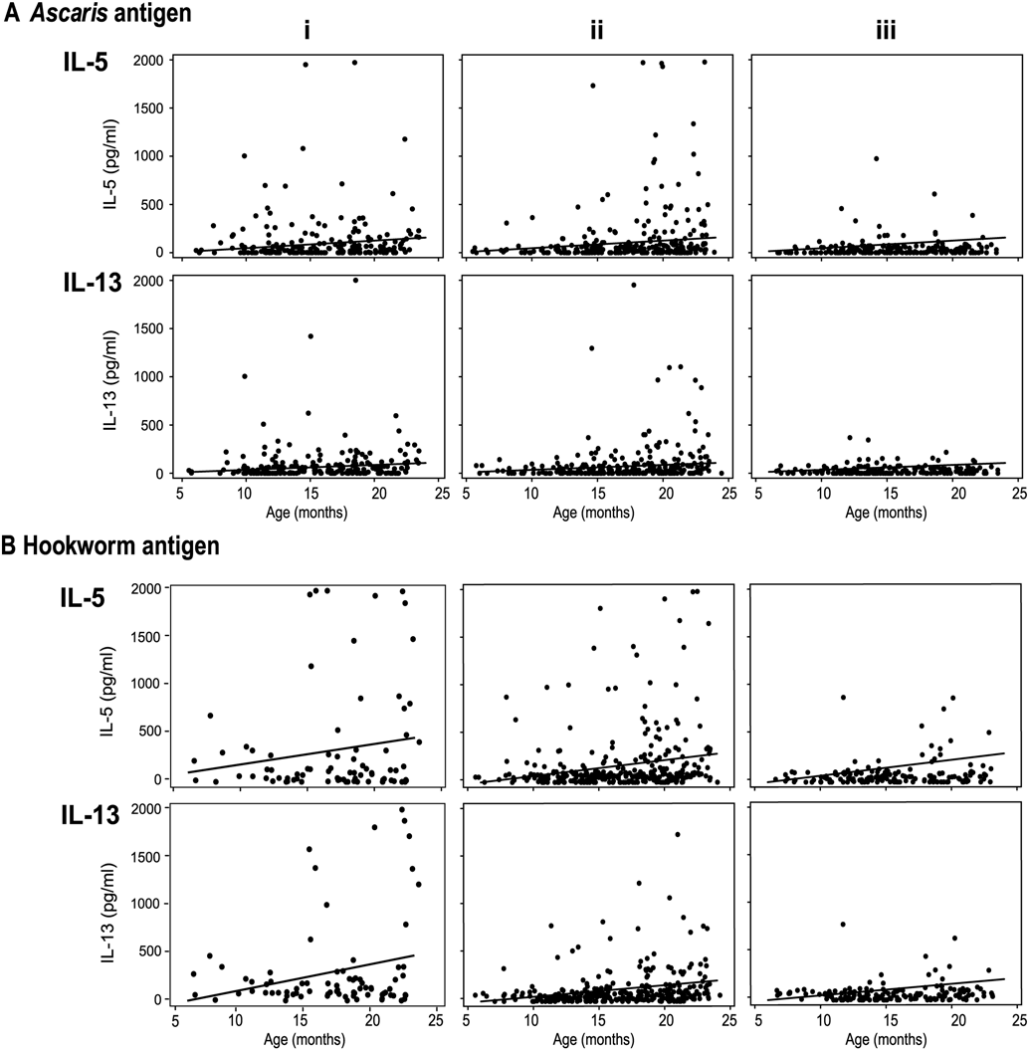
Distribution of IL-5, IL-13 responses to *Ascaris* and hookworm antigens with age by infection status. The lines describe the estimated linear relationship between cytokine responses and age. A) responses to *Ascaris* antigen by (i) all *Ascaris* positive infants, n = 198; Aii) all *Ascaris* negative/other worm positive infants, n = 273; Aiii) worm negative infants, n = 195 and in Bi) all hookworm positive infants, n = 79; Bii) all hookworm negative/other worm positive infants, n = 312; Biii) worm negative infants, n = 164.

Figure 3 shows the levels of Th2 cytokine responses to *Ascaris* and hookworm antigen respectively in relation to age and infection status.

#### *Ascaris* antigen-specific cytokine responses

For *Ascaris*-positive subjects (Figure 3A), there was a positive albeit not significant trend for IL-5 and IL-13 responses with age, whilst these cytokines were significantly positively associated for the *Ascaris* negative/other worm positive group (n = 273, bootstrap repetitions = 500; IL-5 β coeff = 13.11±SE 3.81 95% CI 5.65–20.57 P = 0.001; IL-13 β coeff = 7.86±SE 2.12 95% CI 3.70–12.01 P<0.001) and the any worm positive infants (n = 471, bootstrap repetitions = 1000; IL-5 β coeff = 9.09±SE = 2.82 95% CI 3.56–14.62 P = 0.001; IL-13 β coeff = 5.79±SE = 1.88 95% CI 2.10–9.48 P = 0.002). IL-10 and IFN-γ responses were not significantly associated with age in any of the worm infected groups (data not shown). There were no significant associations between age and any cytokine in worm negative infants or between cytokine response and egg count for *Ascaris* positive infants (data not shown).

#### Hookworm antigen-specific cytokine responses

As seen in Figure 3B, a clear trend with age was seen for IL-5 and IL-13 responses in the hookworm positive group which reached significance for IL-13 (n = 79, bootstrap repetitions = 500 β coeff = 28.18±SE 12.60 95% CI 3.49–52.87 P = 0.025). IL-5 and IL-13 also had significant positive associations with age in any-worm positive (n = 391, bootstrap repetitions = 1000; IL-5 β coeff = 18.04±SE 4.57 95% CI 9.09–26.99 P<0.001; IL-13 β coeff = 14.65±SE 3.62 95% CI 7.54–21.75 P<0.001) and hookworm negative/other worm positive infants (n = 312, bootstrap repetitions = 500; IL-5 β coeff = 16.28±SE 4.59 95% CI 7.29–25.28 P<0.001; IL-13 β coeff = 10.15±SE 2.45 95% CI 5.35–14.95 P<0.001). There was weak but significant correlation between IL-10 and age for the hookworm negative/other worm positive (n = 312, bootstrap repetitions = 500; β coeff = 0.32±SE 0.15 95% CI 0.03–0.61 P = 0.03) and all worm positive groups (n = 391, bootstrap repetitions = 1000; β coeff = 0.32±SE 0.14 95% CI 0.05–0.58 P = 0.018) (data not shown). There were no significant associations between IFN-γ and age in any of the infection groups (data not shown) or between cytokine response and egg count for all hookworm positive infants (data not shown).

#### *Trichuris* antigen-specific cytokine responses

Very few infants made cytokine responses to the *Trichuris* antigens and the levels of response were very low. Although worm positive infants made higher IL-5 and IL-13 responses compared to worm negative infants there was no significant association with age and cytokine concentration to the *Trichuris* antigens in any of the infection groups (data not shown).

### Periodic treatment successfully reduces prevalence and intensity of infection

**Figure 4:**
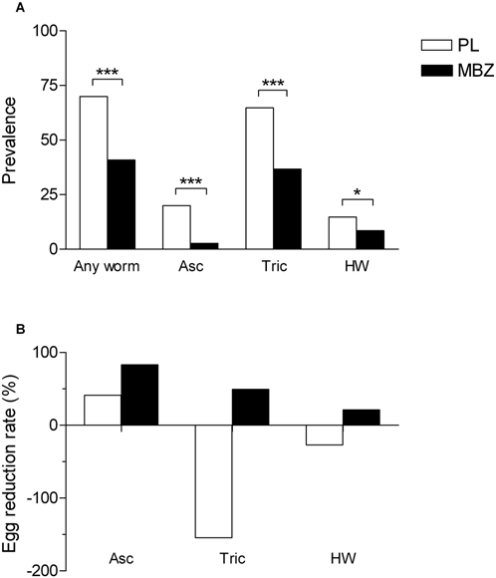
Effects of tri-monthly mebendazole or placebo treatment in infants on parasitology. A. Prevalence of infection after 4 rounds of tri-monthly mebendazole (n = 296) or placebo (n = 307) treatment on any worm positive infants, *Ascaris lumbricoides* positive infants (Asc), *Trichuris trichuria* positive infants (Tric), hookworm positive infants (HW). B. Egg reduction rate after 4 rounds of tri-monthly mebendazole (n = 296) or placebo (n = 307) treatment on *Ascaris lumbricoides* positive infants (Asc), *Trichuris trichuria* positive infants (Tric), hookworm positive infants (HW). * P = 0.01–0.05, *** P<0.001.

The parasitology data following the last round of 3-monthly mebendazole treatment are shown in Figure 4A and 4B. In the mebendazole treated group the prevalence of any worm infection at follow-up (40.9%) was reduced by 42% compared to baseline (70.3%) (z = 7.20 P<0.001, data not shown) and was 41% lower compared to the placebo group (69.9%) (Chi^2^ = 50.7237 P<0.001). In the placebo group there was no significant difference between prevalence at baseline (68.9%) and follow-up (69.9%). The reduced prevalence in the mebendazole compared with the placebo treated infants was also seen when stratified by worm species (*Ascaris* 2.7 vs 19.9% z = 6.6352 P<0.0001, *Trichuris* 36.8 vs 64.7% z = 6.8413 P<0.001, and hookworm 8.4 vs 14.7% z = 2.3954 P = 0.0166). The egg reduction rate followed a similar pattern with mebendazole causing a greater reduction in egg count for *Ascaris* infections, followed by *Trichuris* and then hookworm infections (Figure 4B).

### Effects of treatment on the cytokine response to helminth antigens in all infants: Frequency of responders

#### *Ascaris* antigen-specific cytokine responses

**Figure 5:**
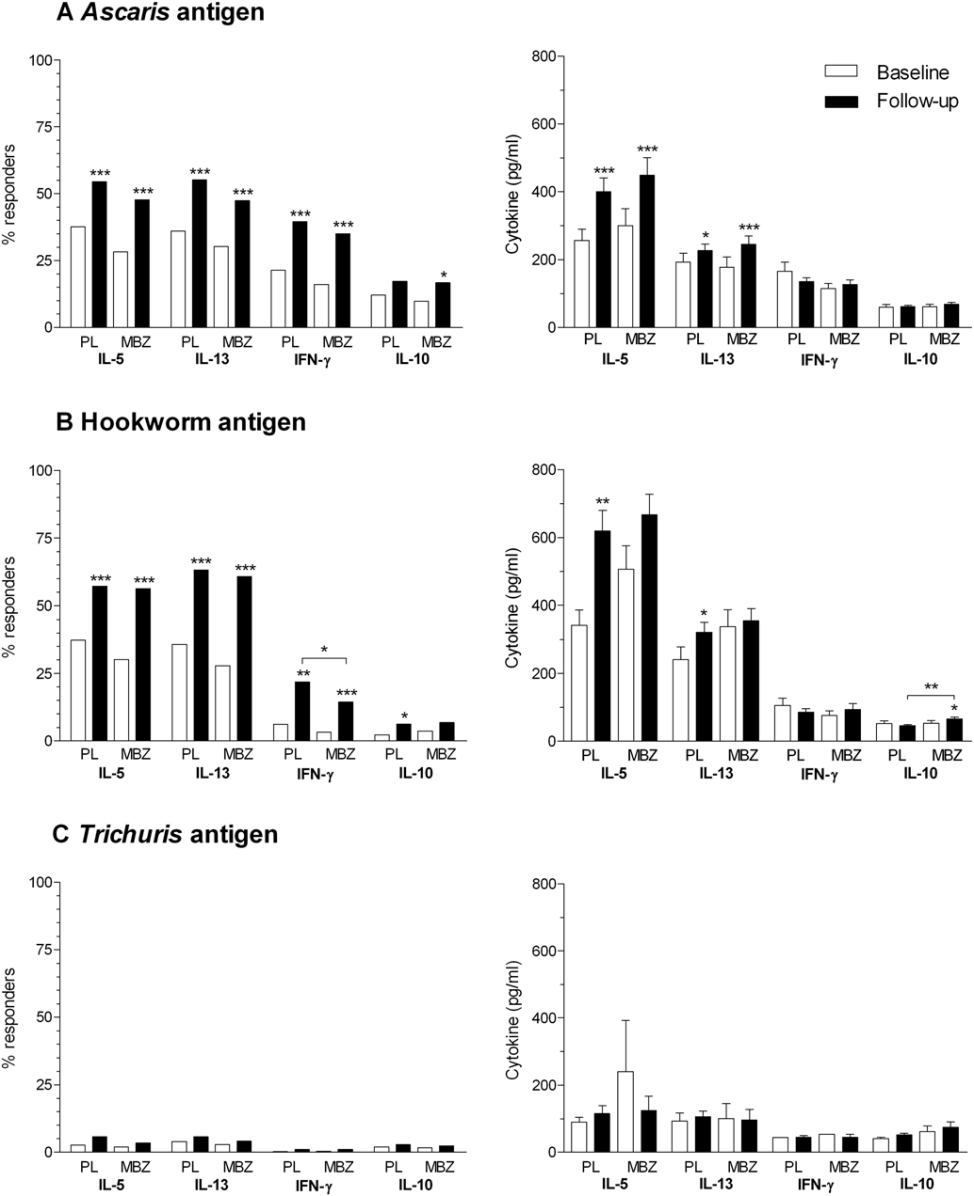
Comparison of cytokine responses at baseline and follow-up in mebendazole or placebo treated infants. Left hand graphs show the percentage of responders (responses >31.24 pg/ml), right hand graphs show the mean (+SEM) response of responders to A) *Ascaris* antigen, B) hookworm antigen C) *Trichuris* antigens for IL-5, IL-13, IFN-γ, and IL-10 in infants treated with placebo (PL) or mebendazole (MBZ) at baseline and follow-up. * P = 0.01–0.05, ** P = 0.001–0.01, *** P<0.001 significantly different from baseline.

As shown in Figure 5A, in both the placebo and mebendazole treated groups, the percentage of responders increased significantly at follow-up compared with baseline for IL-5, IL-13 and for IFN-γ whilst the frequency of IL-10 responders was significantly higher in the mebendazole treated group only. However, anthelminthic treatment during this period had no significant effect on the overall frequency of responders to any of these cytokines.

#### Hookworm antigen-specific cytokine responses

As for *Ascaris*, the percentage of responders to hookworm antigen in both the placebo and mebendazole groups increased for all cytokines from baseline to follow-up but again treatment did not significantly alter the frequency of response except for a modest reduction in the IFN-γ response in the mebendazole treated group (z = 2.36 P = 0.0185) (Figure 5B).

#### *Trichuris* antigen-specific cytokine responses

The percentage of responders to the *Trichuris* antigen was very low at both baseline and follow-up and was similar between the placebo and mebendazole groups at follow-up (Figure 5C).

### Effects of treatment on the cytokine response to helminth antigens in all infants: Mean level of response in relation to treatment in responders

#### *Ascaris* antigen-specific cytokine responses

As for the frequency of response, so the mean levels of cytokine responses in both placebo and mebendazole treated groups increased significantly from baseline to follow-up for IL-5 and IL-13 but treatment had no significant effect on the overall cytokine levels compared with placebo at follow-up (Figure 5A).

#### Hookworm antigen-specific cytokine responses

The level of response to hookworm antigen also showed a tendency to increase between baseline and follow-up for IL-5 and IL-13 although this was only significant in the placebo group (Figure 5B). Again treatment had no significant effects on the response with the exception that the level of IL-10 was higher in the treated group (t = 3.5732 P = 0.001).

#### *Trichuris* antigen-specific cytokine responses

Responses were low and not significantly different between baseline and follow-up or between treated and not treated infants (Figure 5C).

### Blood eosinophilia in infants

**Figure 6:**
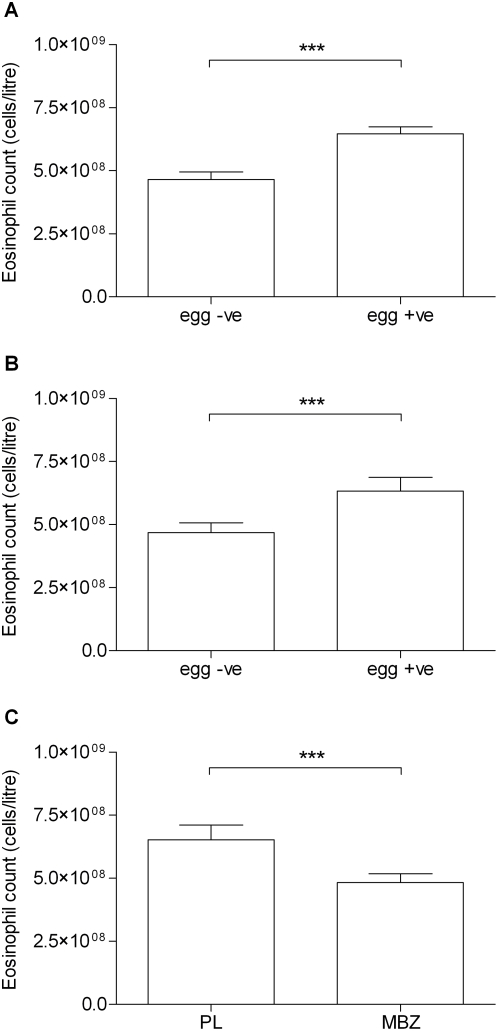
Peripheral blood eosinophil counts at baseline and follow-up and treatment effects. A) worm egg +ve or −ve infants at baseline; B) worm egg +ve or −ve at follow-up, C) MBZ- vs PL-treated children at follow-up. *** P<0.001.

In view of the correlation between worm infection and levels of IL-5, which controls eosinophil production, maturation, migration and persistence in the tissues [49]–[52], the pattern of blood eosinophilia was of interest. As seen in Figure 6, worm positive infants had a significantly higher mean eosinophil count compared to worm negative infants at baseline (Figure 6A) and at follow-up (Figure 6B). Also the mean count was slightly but significantly lower (P = 0.0039) in mebendazole treated infants compared with placebo (Figure 6C). By comparison, basophil counts in infants were minimal (<1%) throughout.

## Discussion

In this study the goals at baseline were i) to establish that GI nematode specific cytokine responses could be measured in infants and ii) to determine the levels and balance of Th1/Th2 cytokines induced by initial exposure of infants to infections with *A. lumbricoides*, hookworm or *T. trichiura*. The prevalence of infection with these helminths in 5–11 month old infants in Pemba at the time of this study was only 26.5% [44] but previous studies in Pemba have shown that the prevalence reaches 90% by the age of 3–5 yr [14]. Transmission on the island is year round and so it is likely that many of the infections detected in the 6–24 month old infants in this study would represent recent primary exposures to infection. Despite this and the fact that the majority of infections were very light, cytokine responses to *Ascaris* and to hookworm antigens could be demonstrated in significant proportions of the infants. Notably cytokines of the Th2 subset predominated amongst responders and there was no evidence of a subset of individuals who made Th1 polarised responses. This is in contrast to primary exposures of mice to the nematode *T. muris* which induces Th2 responses in certain inbred strains but Th1 responses in others [39].

Although *T. trichiura* was a common infection in this age group minimal cytokine responses were seen to *Trichuris* antigen. Low cytokine responses to *Trichuris* antigens have also been reported in some studies of older humans [53],[54] while others have reported higher responses [55],[56]. We do not consider that the failure to detect cytokine responses to *T. trichiura* infection in the infants was due to the use of the heterologous *Trichuris suis* antigen since we showed in preliminary studies with the WBA that this antigen was able to stimulate cytokine production from blood of *T. trichiura* infected Pemban teenagers. Furthermore, we also found that heterologous antigen from *T. muris* (kindly provided by Prof J Bradley, University of Nottingham, UK), which has been shown to induce cytokine responses in older humans in other studies [55],[56] also failed to stimulate cytokine production from our *T. trichiura* infected infants (data not shown). It is possible that the apparently greater sensitization to *Ascaris* and hookworm antigen compared to *Trichuris* is due to the fact that, unlike *Trichuris*, *Ascaris* and hookworms have a larval migratory phase which may have a major role in immune stimulation as was reported in an experimental hookworm infection [35].

The cytokine responses to *Ascaris* and hookworm antigens were significantly positively associated with age at baseline and also consistently increased between baseline and follow-up. These differences between baseline and follow-up were not due to technical differences since there was overlap between the testing of the samples from the baseline and follow-up and validation of all cytokine assays over the course of the study using a pool of positive control supernatant which was included on all plates. Since, the cytokine responses to PHA or PPD did not show this consistent increase at follow-up (data not shown) we conclude that the increased response reflects increased worm exposure over time. The greater frequency and level of Th2 compared with Th1 responses to *Ascaris* and hookworm antigen seen at baseline were maintained over a year of further exposure. Such a Th2 bias is also apparent following prolonged exposure to *Ascaris* and *Trichuris* infections [22],[55] but a more balanced Th1/Th2 cytokine response has been reported in hookworm infection in children and adults [24],[57],[58] although these studies employed purified peripheral blood cell in culture rather than whole blood which may have influenced the cytokine profiles demonstrated.

Amongst the *Ascaris* or hookworm egg positive infants there were a higher proportion of responders to the homologous antigen than amongst egg negative infants or infants with a different species of worm indicating some degree of specificity in the antigen responses to particular worms. However, a proportion of infants lacking *Ascaris* or hookworm infections but harbouring one or both of the other worms also responded to *Ascaris* or hookworm antigens respectively. This could be due to antigen-specific sensitization by prepatent infections in these individuals or to a failure of parasitological detection of infection. However, it may also reflect a degree of cross-reactivity in the responses to the worm antigens as previously suggested [22],[59]. Antigenic cross-reactivity is also supported by the work of Jackson et al 2004 [56] who reported that cytokine responses to somatic *T. trichiura*, *T. muris* and *A. lumbricoides* antigens in WBA were strongly intercorrelated even though the majority of people in the study area had single *T. trichiura* or *A. lumbricoides* infections. Another possible explanation for positive responses in parasitologically negative infants could be prenatal priming to helminth antigens in helminth infected mothers [60].

A proportion of infants who were infected with *Ascaris* and hookworm did not make detectable antigen-specific cytokine responses. This was not correlated with intensity of infection and so does not seem to be due to a sub-threshold level of immune priming. A possible explanation is that the larval phase of infection rather than the persisting egg-laying adult worms may be largely responsible for the cytokine production [35] and so responders may be the more frequently/more recently exposed individuals.

With regard to our starting hypothesis, that the immune response to the worms may contribute to anaemia and wasting malnutrition in infected infants it is clear that the idea of Th1 sensitisation leading to pro-inflammatory cytokines such as TNF-α and IL-6 affecting nutrient metabolism, erythropoiesis and appetite is not supported by this data. The analysis of acute phase proteins and nutritional indicators will be reported elsewhere (manuscript in preparation). It is conceivable that the GI nematode-specific Th2 cytokines demonstrated in infants could be responsible for impaired nutrition due to effects on gut function as demonstrated in mice and pigs [27]–[31] and/or increased nutritional demand due to the generation of immune components. However, we found no evidence that periodic anthelminthic treatment reduced the level of systemic Th2 responses although it again led to reduced anaemia and wasting malnutrition (manuscript in preparation). The only response correlating with worm infection status which was significantly altered by treatment was the decline in peripheral blood eosinophilia. Eosinophil infiltration local to sites of worm infestation has been shown in humans harbouring light *T. trichiura* infections [61] and eosinophils have been implicated in the enteritis induced by zoonotic hookworms [36]. So perhaps eosinophils are involved in mediating gut inflammation and impairing nutrition. Other locally generated responses could impact on gut inflammation and function e.g. helminth-infected infants make more pronounced inflammatory cytokine responses to generic TLR ligands [62]. It should be pointed out that immune responses local to the worms in the gut may differ from recall responses seen in the periphery e.g. in pigs *T. suis* induces a much higher frequency of IL-4 positive cells in ileo-caecal lymph node lymphocytes compared to PBMCs [63]. So reduction in the numbers of worms by chemotherapy may significantly reduce local immunopathological effects in the gut even in the face of unaltered systemic immune responses.

Following the implementation of various helminth control programmes in Pemba Island, the prevalence and intensity of infections in the infants in this study were low and *Trichuris* predominated. Similar studies in areas of higher transmission and with other species predominating would be of interest.

## Supporting Information

Figure S1 Consort flow chart. W+ = worm positive infants, W− = worm negative infants
(0.06 MB TIF)

Figure S2 Number of infants infected by *Ascaris* with age. Worm infection status based on faecal egg examination presented by number of infants infected (Ai & Bi) and as a percentage of each age class within the overall study population (Aii & Bii). A) *Ascaris*±other worms B) *Ascaris* infections only.
(0.58 MB TIF)

Figure S3 Number of infants infected by hookworm with age. Worm infection status based on faecal egg examination presented by number of infants infected (Ai & Bi) and as a percentage of each age class within the overall study population (Aii & Bii). A) hookworm±other worms B) hookworm infections only.
(0.53 MB TIF)

Figure S4 Number of infants infected by *Trichuris* with age. Worm infection status based on faecal egg examination presented by number of infants infected (Ai & Bi) and as a percentage of each age class within the overall study population (Aii & Bii). A) *Trichuris*±other worms B) *Trichuris* infections only.
(0.62 MB TIF)

Figure S5 Distribution of helminth infection intensity by age group. Infection intensity (geometric mean (±95% CI)) based on faecal egg examination presented by age in A) *Ascaris* positive infants only. 6–13 n = 59, 14–18 n = 69, 19–24 n = 56, B) hookworm positive infants only. 6–13 n = 14, 14–18 n = 23, 19–24 n = 26 and C) *Trichuris* positive infants only. 6–13 n = 50, 14–18 n = 113, 19–24 n = 145.
(0.28 MB TIF)

Protocol S1 Protocol for Trial 83988447 (Bickle MS)
(0.11 MB PDF)
