# Supplementary figures and images for: Early Exposure of Infants to GI Nematodes Induces Th2 Dominant Immune Responses Which Are Unaffected by Periodic Anthelminthic Treatment
Source: PLoS Negl Trop Dis. 2009 May 19;3(5):e433. doi: 10.1371/journal.pntd.0000433 (PMC2677666; doi:10.1371/journal.pntd.0000433)

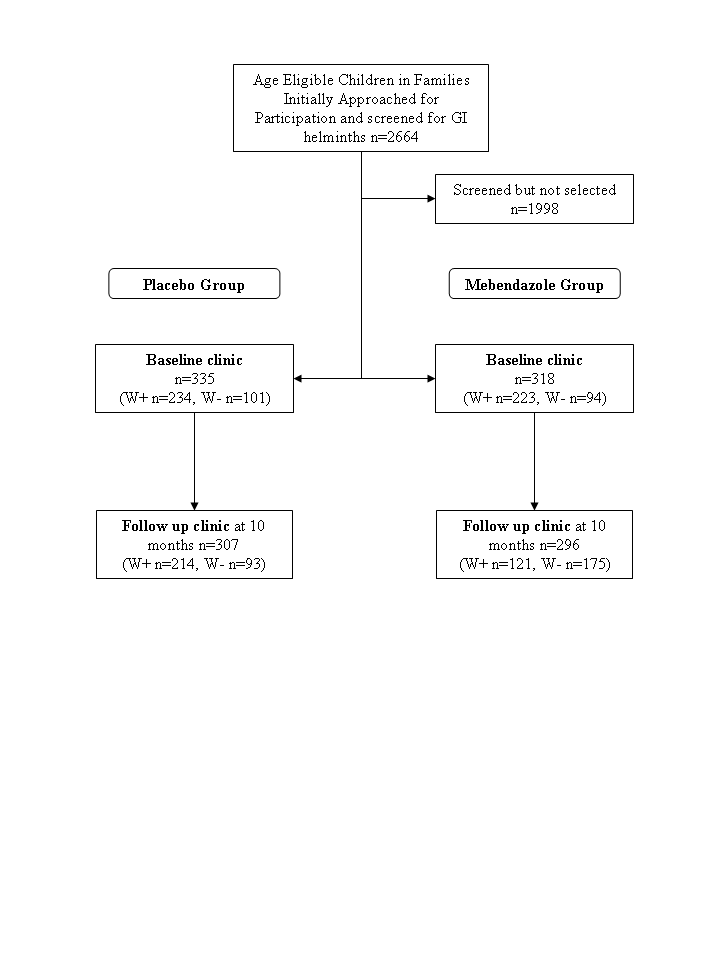

Supplement: Figure S1 — Consort flow chart. W+ = worm positive infants, W− = worm negative infants (0.06 MB TIF) [file pntd.0000433.s001.tif]
