# Supplementary material for: Early Exposure of Infants to GI Nematodes Induces Th2 Dominant Immune Responses Which Are Unaffected by Periodic Anthelminthic Treatment
Source: PLoS Negl Trop Dis. 2009 May 19;3(5):e433. doi: 10.1371/journal.pntd.0000433 (PMC2677666; doi:10.1371/journal.pntd.0000433)
Supplement: Protocol S1 — Protocol for Trial 83988447 (Bickle MS) (0.11 MB PDF) [file pntd.0000433.s006.pdf]

WATOTO BORA STUDY

EFFECT OF EARLY HELMINTH INFECTION ON GROWTH, ANEMIA AND APPETITE

A collaborative project of:

The Public Health Laboratory "Ivo de Carneri"

Investigators: Mahdi Ramsan Mohammed (PI)
Hamad Juma Haji

The Johns Hopkins Bloomberg School of Public Health

Investigators: Rebecca Stoltzfus (PI), now at Cornell University
Jim Tielsch (co-PI)
Benjamin Caballero
Kimberly O'Brien

Cornell University

Investigators: Rebecca Stoltzfus (PI)

The London School of Hygiene and Tropical Medicine

Investigators: Quentin Bickle (PI)
Rosemary Weir
John Raynes
Martin Taylor

1. Study Protocol

Aims Of The Project

Aim 1: To test whether community-based periodic treatment of children 6-23 months old with mebendazole will decrease rates of severe anemia and protein-energy malnutrition by at least 20%.
Aim 2: To determine whether young children infected with intestinal nematode infections exhibit a more pro-inflammatory response to nematode antigens (i.e. characteristic of T Helper 1 [Th1] cells) than age-matched uninfected children.
Aim 3: To test whether mebendazole treatment of infected children reverses this pro-inflammatory response.
Aim 4: To measure intestinal blood loss related to relatively light hookworm infection in young children, and whether intestinal blood loss is reduced after treatment with mebendazole.
Aim 5: To elucidate metabolic mechanisms that could explain the link between a pro-inflammatory cytokine response and anemia and protein-energy malnutrition. Specifically, we aim:

A) To determine whether newly infected children show defects in iron metabolism compared to age-matched uninfected children, in a manner consistent with mechanisms of the "anemia of chronic disease" (i.e. decreased iron absorption, decreased iron incorporation into erythrocytes, suppressed erythropoiesis, and/or shortened red cell life span);
B) To determine whether newly infected children catabolize protein at greater rates than age-matched uninfected children;
C) To determine whether newly infected children are more anorexic than age-matched uninfected children.

Background And Preliminary Data

Malnutrition, measured as low weight-for-age, is one of the strongest known risk factors for child mortality in developing countries. Based on an analysis of data sets from several diverse geographic and socio-cultural locations, 25 and 50% of child deaths between the ages of 1 and 4 years were statistically attributable to malnutrition [1]. Although the risks associated with severe malnutrition are highest, more deaths are attributable to mild-moderate malnutrition, because it is so common. The relative contribution of mild-moderate malnutrition is especially great in sub-Saharan Africa[1]. Severe anemia may also contribute to mortality in children, especially when it coexists with respiratory illness[2;3]. Both protein-energy malnutrition and anemia are also associated with behavioral alterations and delays in child development[4-6].

The causes of childhood malnutrition are multiple. Inadequate dietary intakes of calories and essential nutrients are a major cause[7], but infectious diseases contribute significantly to both anemia and poor growth [8]. The role of *subclinical* infection as a cause of poor growth in young children was hypothesized long ago [9], but its importance in populations has been difficult to demonstrate[10]. The hypothesized pathways are mediated by pro-inflammatory cytokines that suppress appetite[11;12], cause losses of protein[13;14], and raise resting energy expenditure[15]. These processes have been best described in adults with chronic disease, but may be particularly important in young children in developing countries.

The role of subclinical infection or inflammation as a cause of anemia—the so-called anemia of chronic disease[16]—is much better described. This too is mediated by cytokines, which

have been shown to inhibit erythropoietin secretion[17;18] and block its action on the bone marrow[19-21]. Iron deficiency is generally considered the predominant cause of anemia in children in developing countries[22], but the role of subclinical infection as a cause of anemia may be grossly underestimated.

The research we propose to carry out in rural Tanzania is designed to test the hypothesis that intestinal nematode infections in young children contribute significantly to protein-energy malnutrition and anemia through a cytokine-mediated inflammatory response. Geo-helminth infections may be the most common subclinical infection encountered by children in tropical environments. The global prevalence of helminth infections is very high—with 20-30% of the world's children affected[23]. In Zanzibar infection with at least one of the intestinal nematodes (hookworm, *Trichuris trichiura,* or *Ascaris lumbricoides*) is nearly universal by the age of 3 years. The public health motivation for studying these infections is strong, as drugs for treatment are inexpensive and widely available. The biomedical motivation is also compelling, as they can be safely studied through randomized trials. Thus, we believe this research will provide practical guidance to public health programs and will test a novel model for elucidating the role of subclinical infection and inflammation as a cause of malnutrition and anemia in early childhood.

Figure 1. Conceptual Framework

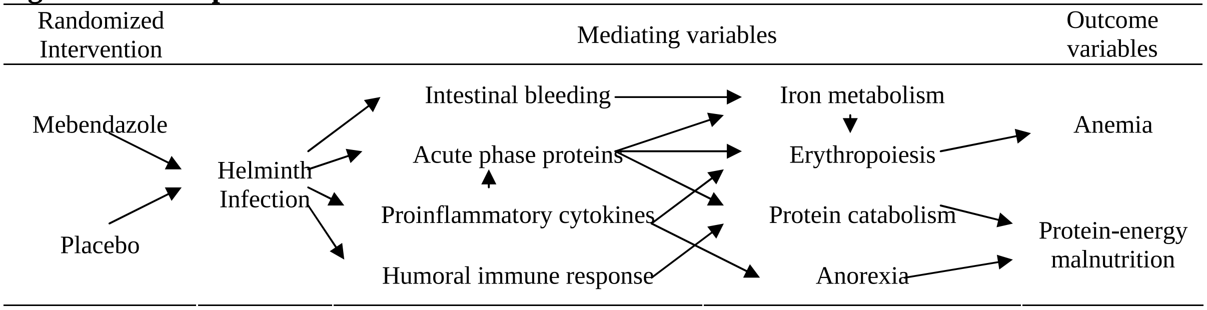

The hypothesis that subclinical intestinal nematode infections may cause wasting malnutrition and the "anemia of chronic disease" in young children is supported by preliminary data from a randomized trial we recently completed in rural Zanzibar (Table 1). A community-based sample of 614 children 6-71 months of age was individually randomized to receive 3-monthly mebendazole (500 mg) or an identical placebo. Our aim was to define the preschool age range in which anthelminthic treatment may be beneficial to child growth and development, and our prior hypothesis was that mebendazole would improve growth and hemoglobin in preschoolers ≥ 3 years old. Contrary to our expectation, we found significant benefits from anthelminthic treatment in children <30 months of age, in whom infection intensities were light but in whom incident infections were common. In this younger age group, mebendazole-treated children showed no improvement in iron status, and their levels of serum ferritin (both an iron storage protein and an acute phase protein) decreased by a large amount. Clinical morbidity symptoms were carefully monitored, and no deleterious effects of mebendazole treatment were observed in any age group. *P. falciparum* is holo-endemic in Zanzibar and clinical cases were treated when detected, but no malaria prevention program was implemented during the study period. The reductions in wasting malnutrition and moderate-severe anemia were large compared to other feasible public health interventions, and may be associated with improvements in child survival and development. However, the statistical significance of the changes was marginal, and the study needs to be repeated in a larger sample of children.

Table 1. Effects of regular mebendazole treatment on rates of low hemoglobin and wasting malnutrition in Pemban children 6-29 months old

	Mebendazole group (n=85)	Placebo group (n=99)	Relative Risk (95% confidence limits)
	Prevalence rates (%)		
Hemoglobin < 90 g/L	24.7	36.8	0.68 (0.43-1.07)
WHZ < -1	22.7	34.8	0.65 (0.40-1.05)
MUAC < 13.5 cm	12.0	22.0	0.53 (0.27-1.05)
Poor appetite	4.7	12.1	0.42 (0.14-1.28)

Stoltzfus RJ, Chwaya HM, Montresor A, Albonico M, Tielsch JM, Savioli L, unpublished data. Children were individually randomized to receive mebendazole (500 mg) or placebo every 3 months for a 12-month period. WHZ=weight-for-height Z score, result is adjusted for baseline value. MUAC=mic-upper-arm circumference, result is adjusted for baseline value and age.

This surprising benefit to young children might be explained by one or both of two things: (1) Children in this age period are at highest risk for the outcomes of severe anemia and wasting malnutrition (well documented facts consistent with our own data) and so are most vulnerable to the deleterious effects of worms, and/or (2) Children during the same period are acquiring first-time helminth infections with acute inflammatory sequelae that are especially detrimental to nutrient metabolism, appetite, and erythropoiesis.

This "incident inflammatory response" might involve: Th1 subset activation and interferon-γ (IFN-γ) production; IFN-γ activation of macrophages to produce such proinflammatory monokines as interleukin-1 (IL-1), interleukin-6 (IL-6), and tumor necrosis factor-α (TNF-α); monokine induction of proinflammatory mediators and acute phase proteins. This novel hypothesis is consistent with the reduction in serum ferritin that we observed when young Pemban children with light infections were treated with mebendazole.

Although chronic gastrointestinal (GI) infections typically stimulate marked Th2 responses characterized by IgE and eosinophilia[24], nothing is known of the characteristics and subsequent development of the acute primary responses in the very young. Studies in rodent models of GI nematode infections have shown that the nature of the cytokine response (Th1 or Th2) to a primary infection is influenced by the host genetic background[25] and also by the dose of infective stages[26]. In this latter study, in the *Trichuris muris* mouse model, a high infective dose of eggs stimulated a strongly Th2-polarized response whereas a light infection stimulated a strong Th1 response (>12-fold increase in IFN-γ). Repeated trickle infection in this murine model eventually led to an increased Th2 response[27]. A switch from Th1 response on primary exposure to a Th2 response following repeated infection has also been shown with *Schistosoma mansoni* infections in baboons. Initial GI nematode infections in young humans will invariably be light and so it seems plausible that infants will make a significant proinflammatory response on first exposure and that this may modulate to a Th2 response with repeated exposure in the absence of treatment. Similar to the differences observed between different mouse strains, human populations also show a marked variation in predisposition to worm infections and host genetic effects on the immune response may similarly influence this[28]. Thus the tendency to produce proinflammatory responses may vary markedly within the population.

Thus, a major focus of this study will be to assess, for the first time, the balance of worm-induced Th1- and Th2-associated cytokines in newly infected infants and how this is influenced by treatment. There is also emerging evidence that helminth (filarial) infections in mothers sensitize for parasite-specific immune responsiveness in neonates[29;30] and that this involves both Th1 and Th2 cytokines, with the pro-inflammatory cytokine IFN-γ prominent[31]. On first exposure to infection, therefore, marked secondary responses may be induced. Whether GI nematode infections

have similar effects is unknown but in Pemba infection rates in women of child-bearing age are very high. In 1995 we conducted a community-based survey of 582 Pemban women of reproductive age, and found that 91% were infected with hookworms[32]. For this reason we will assess maternal helminth infection rates as a potential covariate of infant immune response to infection.

Experimental Design and Methods

We propose to conduct a community-based individually randomized trial of 3-monthly mebendazole treatment to assess the outcomes described in the framework and aims. The study area will be a geographically contiguous group of villages selected for the convenience of sample transport to the laboratory (within about 30 minutes drive by landrover). The PHL is currently conducting a complete population census of Pemba Island, which will allow us to define an area large enough to generate the needed sample size.

A representative sample of 2500 children ages 6-23 months will be screened for helminth infection. All 2500 children will be randomly allocated to treatment with a 3-day course of mebendazole, 100 mg twice daily, or identical placebo. This regimen was chosen to replicate the same drug used in our preliminary trial, but to maximize the efficacy of this drug against hookworms and *T. trichiura*. The study period will be 12 months duration, so that children have their first and final assessments in the same calendar month of the year, thus holding constant any seasonal effects. Anemia and PEM (**Aim 1**) will be assessed in the full sample.

To answer **Aims 2 & 3**, age-matched triplets of children will be selected according to their infection status at baseline. A triplet will be comprised of two infected and one uninfected child of similar age. The two infected children will be matched for species of infection. 250 matched triplets (total n=750) will comprise the Immunology Subsample. We chose 2:1 matching of infected to uninfected, because we are dealing with 3 species of infection and we want to increase our capacity to examine differences by species. Based on our preliminary data, a starting sample of 2500 children will be required to generate at least 500 infected cases. These children will be assessed for immunological parameters at baseline (prior to treatment) and again in the 10th month of the study, one month after the 4th (9-month) treatment round. The post-treatment time was chosen to maximize treatment effect by capturing all 4 rounds of treatment and by timing the assessment one month after the final treatment, when egg counts should be maximally reduced by the mebendazole.

To answer **Aim 4**, a subsample of 300 children will be constructed of hookworm-negative (n=100), light hookworm infections (1-999 epg, n=100) and moderate hookworm infections (≥1000 epg, n=100). These classifications are lower than typically used in school children, and were chosen for children 6-24 month who have lighter infection intensities but whose total body iron store is limited or absent. Therefore small quantities of GI blood loss could have significantly alter their iron balance. Intestinal blood loss will be measured in this subsample at just prior to treatment, and 1 and 12 months post-treatment. This HemoQuant subsample will be drawn from the Immunology Subsample, so that immunologic data will also be available.

To answer **Aims 5A and 5B**, a subsample of 20 newly-infected and 20 uninfected (total n=40) children will be selected from the Immunology Subsample. This number, although small for an epidemiologic study, is large for a metabolic study involving stable isotopes. To our knowledge it is the largest sample size ever involved in a similar protocol. However, many factors may affect the metabolism of these children and it is essential to limit the heterogeneity within this sample to reduce variance unrelated to the factors of interest (i.e. helminth infection status and cytokine response). Thus, we will impose inclusion criteria based on age (12-23 months old), wasting malnutrition (weight-for-height Z scores ≥-1), recent illness (no significant morbidity in the prior 2

weeks, and malaria infection (<1000 parasites/mL). Because these children will be drawn from the age-matched triplets defined for the Immunology study, the 20 children in the newly-infected group and the uninfected groups will be further matched on age within the 1-year-old age category. **Specific Aim 5C** will be addressed using maternal reports of poor appetite, a method that is valid[33] and very simple to implement. Therefore anorexia will be assessed in the full sample of 2500 children just prior to treatment and at 3, 6 and 12 months post-treatment.

Figure 2. Data collection schedule.

Study Month:	0	1	2	3	4	5	6	7	8	9	10	11	12
**FULL SAMPLE** (n = 2500)													
Fecal samples	X												X
Randomized Rx	X			X			X			X			X
Capillary blood[1]	X			X			X			X			X
Appetite	X			X			X			X			X
MUAC	X			X			X			X			X
Mother's milk[2]	X												
**IMMUNOLOGY SUBSAMPLE** (n=750) [3]													
Mother's fecal	X										X		
Venous blood[4]	X										X		
Anthropometry	X										X		
HemoQuant[5]	X										X		

1) Capillary blood sample for determination of hemoglobin and malaria parasite density.
2) Only collected from mothers of children 6-12 months old.
3) Data collected in subsamples are in addition to the data collected for the full sample.
4) Venous blood sample will be used for assays of cytokine secretion in response to antigenic stimulation (whole blood asay), acute phase proteins, immunoglobulins, and iron status indicators.
5) HemoQuant assay will be performed on fecal samples collected for parasitology; no additional samples will be collected.

Justification Of Sample Size

Our starting point for the sample size calculation was the Immunology Subsample. The proposed starting sample size of about 750 children, after 15% attrition, provides 220 children per infection status group, randomly allocated into 2 treatment groups. Thus to compare treatment effects within infection groups, we plan to have about 110 children. Based on the ongoing work of Drs. Taylor, Bickle, and Weir in Nanjing China, where they are using the whole blood assay to assess cytokine reponses to *Schistosoma mansoni*, 100 per group is a minimum number to detect important differences.

To generate this number of age-matched triplets for the Immunology Subsample, about 2500 children will need to be screened for infection. This will in fact be more than enough to address Aim 1. However, we propose to retain the entire group of 2500 children for several reasons. First, we want to retain the representative community-based sample to address Aim 1. The Immunology Subsample will be specifically constructed such that it is no longer representative of the community of young children. We want to make inference about the effects that would be observed if mebendazole treatment were provided to all young children in this environment, since screening for infection status would be impossible to implement in a public health program. Second, it has been our experience in Pemba that once community meetings have been held to inform and motivate participation in a research study, it is difficult to randomly select only some of the children into the actual study. Because participation in the study will confer some benefits to the study subjects and their communities, families resent being excluded from community-based studies. Third, the costs of implementing the Full Sample protocol are relatively small.

The sample size for the HemoQuant Subsample, 100 per infection intensity group, was based on our previous HemoQuant study of hookworm-related blood loss in Pemba school children[34]. In that study we detected significant differences amongst infection intensity groups with 50 children per group, however, the spectrum of infection intensities in school children was much greater. Because infection intensities will be light in these young children, we have doubled our previous sample size to detect smaller blood losses.

The sample size for the metabolic studies was based on the high cost of isotopes and their analysis, and our previous experience conducting studies of this sort. Groups of 10 individuals are typical of most metabolic studies that use stable isotopes. The proposed sample size of 40 children will be large in the published literature; however, the heterogeneity of the children (with regard to nutritional status, malaria, and other recent infections) will be greater than most populations that have been studied.

Assessment Methods For Full Sample

Helminth infection will be assessed by fecal egg counts determined by quantitative formalin ether concentration technique. Presence and intensity of GI nematode infections will be assessed by the Formol ether method on a weighed amount of mixed feces (≈1 g). The eggs present in the whole sediment will be counted in McMaster chambers except if very high numbers of a particular species is present, in which case a known proportion of the graduated slide will be counted.

Anemia will be assessed by hemoglobin concentration using the HemoCue method[35]. When capillary blood samples are assessed (full sample protocol), the third drop of blood will be used[36]. Severe anemia will be defined as hemoglobin <7 g/dL.

Malaria parasite density will be determined by counting parasites against leukocytes in a thick blood film, according to standard methods[37].

Anorexia will be assessed by maternal recall using established techniques[38;39].

PEM will be assessed by weight, length, and arm circumference-for-age[40] following standard protocols.[41]

Additional Assessment Methods For Immunology Subsample

Phlebotomy. Blood (5 mL sample) will be drawn at comparable times of the day into a vacutainer without anti-coagulant. 1 ml will be immediately dispensed into a sterile tube with heparin to be used in the whole blood assay (below). Additional drops will be used to make blood films for total leukocyte and reticulocyte counts, and malariometry. The remaining 2 ml will be allowed to clot and serum will be collected.

Whole Blood Assay to evaluate T cell responses. This assay will be carried out as described previously[42]. Cells will be stimulated with worm antigens (Trichuris, hookworm or Ascaris); the mitogen, PHA; and purified protein derivative (PPD) of Mycobacterium tuberculosis. PPD has been chosen as a positive control antigen as BCG vaccination is given at birth. Supernatants will be harvested at appropriate times for measurement of Interleukin (IL-) 4, 5, 6, 10, 13, TNF-α and IFN-γ. Optimum harvesting time for each cytokine to the antigens will be defined in the study population in project year 1. Cytokines will be measured by quantitative ELISA using commercially available antibody pairs and recombinant cytokine standards (Pharmingen). To control for interplate variation, a positive control supernatant from bulk PHA or LPS stimulated cultures will be used on all plates. IL-4, IL-13, and IL-5 have been chosen as indicators of activation of Th2 type responses, each of which also plays a defined role in the protective immune response against helminth infections[43]. IL-10 is implicated in the suppression of IFN-γ mediated Th1 responses, for example in the early response to S. mansoni infection in humans[44]. IFN-γ has been chosen as a measure of Th1 activation, and for its important role in the induction of TNF-α.

TNF-α and IL-6 are proinflammatory cytokines (which induce acute phase proteins) produced by macrophages following their activation by IFN-γ. TNF-α may play a direct role in regulating Th2 responses to helminths[45], and has a direct effect on cachexia and wasting. All of these cytokines have been measured in whole blood cultures using the techniques developed by Weir et al. (1999 and unpublished) and by others[46]. The utility of measuring cytokine responses in the peripheral blood as an indicator of responses elsewhere in the body has recently been confirmed[47]. Antigens for the whole blood assay will be prepared as follows. Trichuris, hookworm and Ascaris adult worms will be recovered by Quantrel (pyrantel/oxantel) treatment of children and adults in the areas in which the main study will be carried out. Worms will be frozen at –70°C and antigen prepared in culture medium by sonication and centrifugation. Protein will be estimated and the antigen concentration optimized for use in the WBA and specific antibody ELISAs.

Serum acute phase proteins. Inflammation will be assessed by ELISA assay of serum C-reactive protein (CRP), α-1-anti-trypsin (α-1-AT), ferritin and eosinophil cationic protein (ECP) in venous samples. CRP and α-1-AT are acute phase proteins known to suppress erythropoiesis[19;21], ferritin is both an iron storage protein and a positive acute phase protein, and ECP is a marker of eosinophil activity that is elevated in helminth infections[48;49]. CRP and α-1-AT will be assessed by in-house ELISA assay systems based on enzyme-labelled (CRP) and biotinylated (α-1-AT) purified proteins[50]. Ferritin will be measured using the commercial kit made by Ramco, Inc. We will develop an ELISA system for assaying ECP following Reimert et al[51].

Antibody detection. Total IgE, a marker of established infections with many species of helminth, will be measured using the Pharmacia ImmunoCap method [52]. Isotype-specific antibody responses will be assessed by ELISA for IgG, IgG1 (associated with Th1 responses), IgG4 and IgE (both associated with Th2 responses) against hookworm, Ascaris, and Trichuris antigens. The contribution of maternal-derived antibody to that measured in the children's sera may be uncertain but the data will be valuable in conjunction with information on infection presence and intensity.

Iron status will be assessed by measurement of erythrocyte protoporphyrin in a drop of whole blood using a digital hematofluorometer (Aviv Biomedical). Serum ferritin and transferrin receptor will also be measured and will be considered as iron status indicators. However, our prior experience in young Pemban children[53] suggests that serum ferritin mainly reflects the acute phase response, and that transferrin receptor mainly reflects erythropoiesis in this population. In contrast, erythrocyte protoporphyrin was relatively specific to iron deficiency.

Erythropoiesis will be assessed according to the model of Beguin[54], which evaluates erythropoietin concentration, transferrin receptor concentrations and reticulocyte index relative to hemoglobin concentration. Erythropoietin and transferrin receptor concentrations will be measured using commercial ELISA kits. Reticulocyte counts will be made on thin blood films examined microscopically.

Morbidity in the week prior to each immunology assessment will be assessed by maternal recall using local terms for acute symptoms.

Additional Assessment Methods For HemoQuant Subsample

Intestinal bleeding will be assessed by the HemoQuant assay[55;56]. This assay measures the quantity of heme excreted per gram of feces. In subjects who consume little red meat intake (like young Pemban children), this is an accurate measure of intestinal blood loss.

Additional Assessment Methods For Metabolic Subsample

The sequence of the metabolic studies is shown in Table 3. Protein catabolism, iron absorption and iron incorporation into red cells will be assessed before mebendazole treatment. Pre-treatment measures in newly infected and uninfected children will allow us to test cross-sectionally for

metabolic changes associated with new infections. We will also assess body composition and plasma volume. This is necessary to estimate body pool sizes, which may differ from normal assumptions because of the chronic mild malnutrition and subclinical infections in Pemban children. One month post-treatment we will reassess protein catabolism, and assess red cell survival rates. Post-treatment values will allow us to assess whether mebendazole reduces protein catabolism or alters red cell life span.

Figure 3. Summary of metabolic studies

	**Time relative to mebendazole treatment**		
	**2 weeks pre-treatment**	**On day of treatment**	**1 month post-treatment**
**Iron metabolism**	Administration of iron isotopes	Assessment of iron absorption and incorporation into red cells	Assessment of red cell survival
**Protein catabolism**	Pre-treatment protein turnover study		Post-treatment protein turnover study
**Body composition and plasma volume**	Isotopic dilution; Sodium bromide dilution		

<u>General procedures.</u> Infants will be brought to an outpatient clinical facility at the Pemba PHL accompanied by an adult relative. The atmosphere will be designed to provide comfort and entertainment for the infants, mothers and any siblings who come along. A medical doctor will be present throughout every protocol, which will be implemented by experienced pediatric nurses. Appropriate incentives will be given to families who participate to compensate their time and inconvenience.

<u>Iron metabolism protocol.</u> Iron absorption and red cell iron incorporation will be measured by administering oral (^{57}Fe) and intravenous (^{58}Fe) stable iron isotopes to each infant. Blood samples will be taken 2 weeks post-dosing to measure the enrichment of these tracers in red blood cells. The enrichment of isotopes in this sample will allow us to measure both iron absorption and the degree of red cell iron incorporation. An additional blood sample will be taken 4 weeks post-treatment to measure potential differences in the rate of erythropoiesis or red cell destruction between infection groups in children treated with placebo (i.e. newly infected vs. uninfected, n=10 per group) or between treatment groups in newly infected children (n=10 per group).

At the 2-week pre-treatment visit, each infant will be given an oral dose of NaBr (1.5 g/kg) and ^{57}Fe (7 mg, as ferrous sulfate). Infants will then be allowed to nurse and will be fed a standard meal 1 hour post-dosing. The majority of prior studies in infants have estimated red blood cell iron incorporation at 90% in infants. This assumption simplifies the studies and allows them to done using only a single oral iron isotope[57;58]. However, the degree of iron incorporation into red cells may be altered in these infants due to subclinical infections. We will therefore directly measure the degree of iron incorporation into red cells by administering a second intravenous iron isotope. An intravenous dose of ^{58}Fe (0.6 mg as ferrous citrate) will be administered over 5 minutes. Blood volume assumptions are also necessary. Most studies estimate blood volume at 65-80 mL/kg. Information obtained from the deuterium (body composition study, see below) and NaBr tracer dosing will allow us to directly measure extracellular fluid volume and total body water and reduces the assumptions necessary for making calculations about iron metabolism. A venous blood sample will be taken 4 hours after the oral dosing to measure NaBr concentration.

At the treatment visit, a 5 mL blood sample will be drawn by venipuncture. One mL whole blood will be digested using ultrapure acids. Iron will be extracted using cation-exchange chromatography. The eluent will be dried and the iron residue will be dissolved in nitric acid. Iron isotope ratios will be determined using magnetic sector thermal ionization mass spectrometry (Finnigan MAT 261, Bremen, FRG). These methods have been previously described in detail.[57-59]

Few data have addressed the impact of parasitic infections on the lifespan of erythrocytes in infants and the timing of maximal iron incorporation into red cells. Studies have indicated that the lifespan of the erythrocyte is markedly lower in infants than adults[60] and a recent study has found alterations in the time course of iron incorporation into erythrocytes in infants[61]. An additional blood sample is already planned at the 1 month post-treatment visit for immunology and protein turnover studies. We will also analyze the enrichment of iron isotopes in these blood samples to compare the stability of the tracer enrichments between infection and treatment groups.

Protein turnover study. Protein catabolism will be assessed by measuring oxidation of leucine, an indispensable amino acid, labelled with a stable tracer[62]. The fraction of leucine flux that is oxidized is constant under normal conditions, but increases markedly with protein breakdown. The protocol consists of two sequential oral tracer infusions: (1) Measurement of CO_2 production: bicarbonate tracer ($^{13}CO_3NaH$ (99% APE, Cambridge Isotopes, MA) will be administered as a priming oral dose of 0.4 mg/kg, followed by a 3-hour constant oral infusion of 0.4 mg/kg/hr, administered in three equal aliquots every 20 minutes[62;63]. (2) Measurement of leucine oxidation: upon completion of the bicarbonate infusion, the leucine tracer infusion will start. L-[1-^{13}C]-Leucine (95% APE, Cambridge Isotopes, MA) will be administered as a priming dose of 2 mg/kg, followed by a 4-hour constant oral infusion of 2 mg/kg/hr for 4 hours. Doses will be given in 20-minute aliquots[64;65]. During the infusion study children will be fed at hourly intervals. Intake of breast milk will be determined by test weighing. Feeding will approximate the pro-rated calculated 24-hour energy requirement for actual weight. Breath air (15 mL) will be sampled for isotopic enrichment by asking the child to blow into a small balloon.

Body composition study. Body composition will be determined by isotopic dilution, using deuterium oxide[66]. A fixed, 20 g oral dose of $^{2}H_2O$ (99.5APE, Cambridge Isotopes, MA) will be administered at the pre-treatment visit at the same time the NaBr is given. Saliva samples will be collected at 3 and 3.5 hours post-dose, for measurement of isotopic enrichments.

Data Analysis

Data will be entered using an Oracle-based data management system. Data analysis will be directed by Dr. Tielsch, with full participation of the research team to interpret and publish findings. Primary analyses will compare relative risks (Aim 1) or means (Aims 2-5) between groups defined by pre-treatment infection status or treatment group, according to the specific aims.

Collaboration

The collaboration between The Johns Hopkins University and the Ministry of Health of Zanzibar has been ongoing since 1994. The focus has been epidemiologic research on the relations between parasitic infections, anemia and malnutrition. The London School of Hygiene and Tropical Medicine (LSHTM, Dr. Bickle, Prof Taylor) is currently collaborating with the PHL to investigate whether Zanzibari school children are acquiring resistance to mebendazole after more than a decade of mass treatment with that drug. They are also collaborating with Nanjing Medical University to study cytokine and antibody responses and resistance to infection with *Schistosoma japonicum* in humans. This proposal unites the nutrition and epidemiology expertise of JHU with infectious disease immunology expertise of the Immunology Unit at LSHTM and the applied health research experience and needs of the Pemba PHL. Each contribution is essential. In June 2000, Ministry of Health of Zanzibar opened the Pemba PHL to serve as a semi-autonomous research body of the MOH. The proposal is designed to equip the PHL with training and technology related to epidemiology, parasitology, and metabolism.

Training And Technology Transfer

Three forms of training are integrated into the research plan. The first is MSc training at an African Institution for one Zanzibari scientist, for whom parts of this research will comprise his/her thesis research. Second, 2 senior local scientists (Dr. Chwaya, Dr. Mohammed, and the MSc trainee) will participate in the 3-week Summer Epidemiology Course offered by JHU in project years 1, 2, and 3. This will allow each one to take a total of 6 short intensive courses (2 per year) in epidemiology and biostatistics. Third, 2-person teams from the Immunology and Parasitology Departments of the PHL will receive practical training on assessment techniques both at the PHL and in LSHTM laboratories. The foci of the training will be the whole blood assay under the direction of Drs. Bickle and Weir, and measurement of acute phase proteins under the direction of Dr. Raynes. Practical training in each of these areas will include two parts: First, a 3-week trip to the US or UK laboratory to learn the method, followed by a visit of the UK or US scientist to the PHL to supervise the establishment of the method in the PHL. In addition to training of personnel, this proposal will provide the Pemba PHL with key laboratory equipment that will serve not only this research plan, but a wide variety of research endeavors in years to come. This equipment is detailed on page 27. The research plan is designed so that all laboratory work will be conducted at the Pemba PHL, with the exception of the analysis of stable isotopes by mass spectrometry which requires very specialized and expensive instruments.

Study Limitations

Young Pemban children are exposed to holo-endemic malaria transmission, a "nuisance" factor that may influence the immunological and metabolic studies. We will assess malaria parasite density at each blood draw and consider it as a covariate in our analyses. *Schistosoma haematobium* is also endemic in Pemba; however we expect these young children will have very limited exposure. Our strongest tool is the randomized intervention design. If we observe differences between treatment groups we can make causal inference about the specific role of helminth infections. Our preliminary data suggest that effects of anthelminthic treatment can be detected and may be large, even in the presence of other concurrent parasitic infections.

1. Pelletier DL. Relationships between child anthropometry and mortality in developing countries: implications for policy, programs, and future research. Ithaca: Cornell University, 1991.

2. Lackritz EM, Campbell CC, Ruebush TK, Hightower AW, Wakube W, Steketee RW *et al*. Effect of blood transfusion on survival among children in a Kenyan hospital [see comments]. *Lancet* 1992;**340**:524-8.

3. Lackritz EM, Hightower AW, Zucker JR, Ruebush TK, Onudi CO, Steketee RW *et al*. Longitudinal evaluation of severely anemic children in Kenya: the effect of transfusion on mortality and hematologic recovery. *AIDS* 1997;**11**:1487-94.

4. Lozoff B. Behavioral alterations in iron deficiency. *Advances In Pediatrics* 1988;**35**:331-59.

5. Grantham-McGregor SM,.Fernald LC. Nutritional deficiencies and subsequent effects on mental and behavioral development in children. *Southeast Asian J Trop.Med.Public Health* 1997;**28 Suppl 2**:50-68.

6. Vazir S, Naidu AN, Vidyasagar P. Nutritional status, psychosocial development and the home environment of Indian rural children. *Indian Pediatr* 1998;**35**:959-66.

7. Martorell R, Habicht J-P. Growth in early childhood in developing countries. In Falkner F, Tanner JM, eds. *Human Growth*, pp 241-62. New York: Plenum Press, 1986.

8. Martorell R, Habicht J-P, Yarbrough C, Lechtig A, Klein RE, Western D. Acute morbidity and physical growth in rural Guatemalan children. *Am J Dis Child* 1975;**129**:1296-301.

9. Beisel WR. Resume of the discussion concerning the nutritional consequences of infection. *Am J Clin Nutr* 1977;**30**:1294-300.

10. Waterlow J. Protein-energy malnutrition: challenges and controversies. *Proc Nutr Soc India* 1991;**37**:59-86.

11. Langhans W,.Hrupka B. Interleukins and tumor necrosis factor as inhibitors of food intake. *Neuropeptides* 1999;**33**:415-24.

12. Plata-Salaman CR. Cytokine-induced anorexia. Behavioral, cellular, and molecular mechanisms. *Ann.N.Y.Acad.Sci.* 1998;**856**:160-70.

13. Reeds PJ, Fjeld CR, Jahoor F. Do the differences between the amino acid compositions of acute-phase and muscle proteins have a bearing on nitrogen loss in traumatic states? *J Nutr* 1994;**124**:906-10.

14. Solomons NW, Mazariegos M, Vettorazzi C, Valdez C, Grazioso C, Romero-Abal ME *et al*. Growth faltering, protein metabolism and immunostimulation: new speculations on the nature of the relationship with notes from observations and analyses in Guatemala. *Application of stable isotope tracer methods to studies of amino acid, protein and energy metabolism in malnourished populations of developing countries.*, pp 115-26. Vienna: IAEA, 1994.

15. Roubenoff R, Roubenoff RA, Cannon JG, Kehayias JJ, Zhuang H, Dawson-Hughes B *et al*. Rheumatoid cachexia: cytokine-driven hypermetabolism accompanying reduced body cell mass in chronic inflammation. *J Clin Invest* 1994;**93**:2379-86.

16. Weiss G. Iron and anemia of chronic disease. *Kidney Int.Suppl* 1999;**69**:S12-S17.

17. Jelkmann W,.Hellwig-Buergel T. Tumor necrosis factor p55 receptor (TNF-RI) mediates the in vitro inhibition of hepatic erythropoietin production. *Exp.Hematol.* 1999;**27**:224-8.

18. Jelkmann W. Proinflammatory cytokines lowering erythropoietin production. *J Interferon Cytokine Res* 1998;**18**:555-9.

19. Nordstrom D, Lindroth Y, Marsal L, Hafstrom I, Henrich C, Rantapaa-Dahlqvist S *et al*. Availability of iron and degree of inflammation modifies the response to recombinant human erythropoietin when treating anemia of chronic disease in patients with rheumatoid arthritis. *Rheumatol.Int.* 1997;**17**:67-73.

20. Davis D, Charles PJ, Potter A, Feldmann M, Maini RN, Elliott MJ. Anaemia of chronic disease in rheumatoid arthritis: in vivo effects of tumour necrosis factor alpha blockade. *Br.J Rheumatol.* 1997;**36**:950-6.

21. Graziadei I, Gaggl S, Kaserbacher R, Braunsteiner H, Vogel W. The acute-phase protein alpha 1-antitrypsin inhibits growth and proliferation of human early erythroid progenitor cells (burst-forming units-erythroid) and of human erythroleukemic cells (K562) in vitro by interfering with transferrin iron uptake. *Blood* 1994;**83**:260-8.

22. Levin HM, Pollitt E, Galloway R, McGuire J. Micronutrient deficiency disorders. In Jamison DT, Mosley WH, eds. *Disease control priorities in developing countries*, Oxford University Press (World Bank): New York, 1993.

23. Warren KS, Bundy DAP, Anderson RM, Davis AR, Henderson DA, Jamison DT *et al*. Helminth infection. In Jamison DT, Mosley WH, Measham AR, Bobadilla JL, eds. *Disease control priorities in developing countries*, pp 131-60. New York: Oxford University Press, 1993.

24. Pritchard DI. The survival strategies of hookworms. *Parasitology Today* 1995;**11**:255-9.

25. Else KJ,.Wakelin D. The effects of H-2 and non-H2 genes on the expulsion of the nematode *Trichuris muris* from inbred and congenic strains of mouse. *Parasitology* 1988;**96**:543-50.

26. Bancroft AJ, Else KJ, Grencis RK. Low-level infection with Trichuris muris significantly affects the polarization of the CD4 response. *Eur J Immunol.* 1994;**24**:3113-8.

27. Grencis RK. T cell and cytokine basis of host variability in response to intestinal nematode infections. *Parasitology* 1996;**112 Suppl**:S31-S37.

28. Bundy DAP. Epidemiology and transmission of intestinal helminths. In Farthing MJC, Keusch GT, Wakelin D, eds. *Enteric Infection 2. Intestinal Helminths.*, pp 5-24. London: Chapman & Hall, 1995.

29. King CL, Malhotra I, Mungai P, Wamachi A, Kioko J, Ouma JH *et al*. B cell sensitization to helminthic infection develops in utero in humans. *J Immunol.* 1998;**160**:3578-84.

30. Malhotra I, Ouma J, Wamachi A, Kioko J, Mungai P, Omollo A *et al*. In utero exposure to helminth and mycobacterial antigens generates cytokine responses similar to that observed in adults. *J Clin Invest* 1997;**99**:1759-66.

31. Soboslay PT, Geiger SM, Drabner B, Banla M, Batchassi E, Kowu LA *et al*. Prenatal immune priming in onchocerciasis-onchocerca volvulus-specific cellular responsiveness and cytokine production in newborns from infected mothers. *Clin Exp.Immunol.* 1999;**117**:130-7.

32. Stoltzfus RJ, Dreyfuss ML, Chwaya HM, Albonico M. Hookworm control as a strategy to prevent iron deficiency. *Nutr Rev* 1997;**55**:223-32.

33. Brown KH, Peerson JM, Lopez de Romana G, Creed de Kanashiro H, Black RE. Validity and epidemiology of reported poor appetite among Peruvian infants from a low-income, periurban community. *Am J Clin Nutr* 1995;**61**:26-32.

34. Stoltzfus, R. J., Yip, R., Chwaya, H. M., Alawi, K. S., Albonico, M., Schulze, K. J., Tielsch, J., and Savioli, L. Quantitation of hookworm blood loss and iron status (abstract). FASEB J . 1995.
Ref Type: In Press

35. von Schenck H, Falkensson M, Lundberg B. Evaluation of "HemoCue," a new device for determining hemoglobin. *Clin Chem* 1986;**32**:526-9.

36. Morris SS, Ruel MT, Cohen RJ, Dewey KG, de la BB, Hassan MN. Precision, accuracy, and reliability of hemoglobin assessment with use of capillary blood. *Am J Clin Nutr* 1999;**69**:1243-8.

37. Trape JF. Rapid evaluation of malaria parasite density and standardization of thick smear examination for epidemiological investigations. *Transactions of the Royal Society of Tropical Medicine and Hygiene* 1985;**79**:181-4.

38. Brown KH, Peerson JM, Lopez dR, de Kanashiro HC, Black RE. Validity and epidemiology of reported poor appetite among Peruvian infants from a low-income, periurban community. *Am J Clin Nutr* 1995;**61**:26-32.

39. Cohen RJ, Rivera LL, Canahuati J, Brown KH, Dewey KG. Delaying the introduction of complementary food until 6 months does not affect appetite or mother's report of food acceptance of breast-fed infants from 6 to 12 months in a low income, Honduran population. *J Nutr* 1995;**125**:2787-92.

40. World Health Organization Expert Committee. Physical status: the use and interpretation of anthropometry. WHO Technical Report Series No. 854. Geneva: World Health Organization, 1995.

41. Callaway CW, Chumlea WC, Bouchard C, Himes JH, Lohman TG, Martin AD *et al*. Circumferences. In Lohman TG, Roche AF, Martorell R, eds. *Anthropometric standardization reference manual.*, pp 39-54. Champaign: Human Kinetics Books, 1988.

42. Weir RW, Brennan PJ, Butlin CR, Dockrell HM. Use of a whole blood assay to evaluate in vitro T cell responses to new leprosy skin test antigens in leprosy patients and health subjects. *Clin Exp.Immunol.* 1999;**116**:263-9.

43. McKenzie GJ, Bancroft A, Grencis RK, McKenzie AN. A distinct role for interleukin-13 in Th2-cell-mediated immune responses. *Curr.Biol* 1998;**8**:339-42.

44. Montenegro SM, Miranda P, Mahanty S, Abath FG, Teixeira KM, Coutinho EM *et al*. Cytokine production in acute versus chronic human Schistosomiasis mansoni: the cross-regulatory role of interferon-gamma and interleukin- 10 in the responses of peripheral blood mononuclear cells and splenocytes to parasite antigens. *J Infect.Dis* 1999;**179**:1502-14.

45. Artis D, Humphreys NE, Bancroft AJ, Rothwell NJ, Potten CS, Grencis RK. Tumor necrosis factor alpha is a critical component of interleukin 13- mediated protective T helper cell type 2 responses during helminth infection. *J Exp.Med.* 1999;**190**:953-62.

46. de Groote D, Geveart Y, Lopez M, Gathy R, Fauchet F, Dehart I *et al*. Novel method for the measurement of cytokine production by a one-stage procedure. *J Immunol.Methods* 1993;**163**:259-67.

47. Taylor MD, Betts CJ, Else KJ. Peripheral cytokine responses to Trichuris muris reflect those occurring locally at the site of infection. *Infect.Immun.* 2000;**68**:1815-9.

48. Tischendorf FW, Brattig NW, Buttner DW, Pieper A, Lintzel M. Serum levels of eosinophil cationic protein, eosinophil-derived neurotoxin and myeloperoxidase in infections with filariae and schistosomes. *Acta Trop.* 1996;**62**:171-82.

49. Vennervald BJ, Reimert CM, Ouma JH, Kilama WL, Deelder AM, Hatz C. Morbidity markers for Schistosoma haematobium infection. *Trop.Geogr.Med.* 1994;**46**:239-41.

50. Gillespie SH, Dow C, Raynes JG, Behrens RH, Chiodini PL, McAdam KP. Measurement of acute phase proteins for assessing severity of Plasmodium falciparum malaria. *J Clin Pathol.* 1991;**44**:228-31.

51. Reimert CM, Venge P, Kharazmi A, Bendtzen K. Detection of eosinophil cationic protein (ECP) by an enzyme-linked immunosorbent assay. *J Immunol.Methods* 1991;**138**:285-90.

52. Pritchard DI, Quinnell RJ, Walsh EA. Immunity in humans to Necator americanus: IgE, parasite weight and fecundity. *Parasite Immunol.* 1995;**17**:71-5.

53. Stoltzfus, R. J., Chwaya, H. M., Montresor, A., Albonico, M., Savioli, L., and Tielsch, J. Malaria, hookworms and recent fever are related to anemia and iron status indicators in 0- to 5-y old Zanzibari children and these relationships change with age. Journal of Nutrition . 2000.
Ref Type: In Press

54. Beguin Y, Clemons GK, Pootrakul P, Fillet G. Quantitative assessment of erythropoiesis and functional classification of anemia based on measurements of serum transferrin receptor and erythropoietin. *Blood* 1993;**81**:1067-76.

55. Ahlquist DA, McGill DB, Schwartz S, Taylor WF, Ellefson M, Owen RA. HemoQuant, a new quantitative assay for fecal hemoglobin. Comparison with Hemoccult. *Annals of Internal Medicine* 1984;**101**:297-302.

56. Stoltzfus RJ, Albonico M, Chwaya HM, Savioli L, Tielsch J, Schulze K *et al*. HemoQuant determination of hookworm-related blood loss and its role in iron deficiency in African children. *Am J Trop Med Hyg* 1996;**55**:399-404.

57. Abrams SA, WenJ.P., Stuff JE. Absorption of calcium, zinc, and iron from breast milk by five- to seven-month old infants. *Pediatr Res* 1997;**41**:384-90.

58. Abrams SA, O'Brien KO, Wen J, Liang LK, Stuff JE. Absorption by 1-year-old children of an iron supplement given with cow's milk or juice. *Pediatr Res* 1996;**39**:171-5.

59. Abrams SA, Wen J, O'Brien KO, Stuff JE, Liang LK. Application of magnetic sector thermal ionization mass spectrometry to studies of erythrocyte iron incorporation in small children. *Biological Mass Spectrometry* 1994;**23**:771-5.

60. Pearson HA. Life-span of the fetal red blood cell. *J Pediatr* 1967;**70**:166-71.

61. Fomon SJ, Serfass RE, Nelson SE, Rogers RR, Frantz JA. Time course of and effect of dietary iron level on iron incorporation into erythrocytes by infants. *J Nutr* 2000;**130**:541-5.

62. Mazariegos, M., Vettorazzi, C., Caballero, B., Fjeld, C. R., Solomons, N. W., and Jahoor, F. Rates of [1-13C] leucine oxidation in Guatemalan children with chronic immunostimulation. FASEB J 10, 474-474. 1996.
Ref Type: Abstract

63. Armon Y, Cooper DM, Springer C. Oral [13C]bicarbonate measurement of CO2 stores and dynamics in children and adults. *J Appl Physiol* 1990;**69**:1759-60.

64. Beaufrere B, Putet G, Pachiaudi C, Salle B. Whole body protein turnover measrued with 13C-leucine and energy expenditure in preterm infants. *Pediatr Res* 1990;**20**:147-52.

65. Bier DM. The use of stable isotopes in metabolic investigation. In Alberti KGMM, Home PD, Taylor R, eds. *Bailliere's Clinical Endocrinology and Metabolism*, pp 817-35. London: Bailliere Tindall, 1987.

66. Guo S, Roche AF, Houtkooper L. Fat-free mass in children and young adults predicted from bioelectric impedance and anthropometric variables. *Am J Clin Nutr* 1989;**50**:435-43.
